# Pain Perception and Psychoemotional Responses Across Different Scaling Technologies: A Comparative Pilot Clinical Study

**DOI:** 10.3390/dj13120597

**Published:** 2025-12-12

**Authors:** Nelsi Carmina Turturica, Mindra E. Badea, Vlad I. Bocanet, Radu Chifor, Iulia C. Badea

**Affiliations:** 1 Department of Preventive Dental Medicine, Faculty of Dental Medicine, Iuliu Hatieganu University of Medicine and Pharmacy, 400012 Cluj-Napoca, Romania; turturica_nelsi_carmina@elearn.umfcluj.ro (N.C.T.); mebadea@umfcluj.ro (M.E.B.); iulia.badea@umfcluj.ro (I.C.B.); 2 Faculty of Machine Building, Technical University of Cluj-Napoca, 28 Memorandumului St., 400114 Cluj-Napoca, Romania; vlad.bocanet@tcm.utcluj.ro; 3 Department of Oral Rehabilitation, Faculty of Dental Medicine, Iuliu Hatieganu University of Medicine and Pharmacy, 400012 Cluj-Napoca, Romania

**Keywords:** dental anxiety, pain perception, patient comfort, patient-reported outcome measures, periodontal diseases, psychological distress, self-esteem, ultrasonic scaling

## Abstract

**Background/Objectives**: Scaling is central to non-surgical periodontal therapy; however, it is often perceived as uncomfortable, particularly in periodontitis. Psychological distress may amplify pain and reduce adherence. This comparative pilot study assessed whether ultrasonic technology influences patient-reported pain and psychoemotional status while quantifying the contribution of the periodontal condition and baseline psychological factors. **Methods**: A monocentric split-mouth design enrolled 42 adults (21 with stage I–III, grade B periodontitis; 21 periodontally healthy). Maxillary scaling was performed with Device nr.1 and mandibular scaling was performed with Device nr.2, and no anesthesia was used. Pain was measured immediately post-procedure using the Short-Form McGill Pain Questionnaire (SF-MPQ; sensory and affective subscales). Psychological status was assessed pre- and post-session with the Kessler Psychological Distress Scale (K10) and the Rosenberg Self-Esteem Scale (RSES). Construct validity was examined via exploratory factor analysis. The Mann–Whitney *U*, Wilcoxon signed-rank, Spearman’s *ρ*, and Cliff’s *δ* were applied (*α* = 0.05). **Results**: The overall pain was low. Between devices, sensory pain did not differ, whereas affective pain was modestly lower with the “No Pain” device (*p* = 0.017). Periodontitis was the dominant determinant of pain: higher sensory (*U* = 509.00, *p* = 0.0004; *δ* = 0.42) and affective scores (*U* = 290.00, *p* < 0.0001; *δ* = 0.67) occurred irrespective of device, while device-related effects were negligible (sensory *δ* = −0.03) to small (affective *δ* = 0.27). Somatic distress correlated with affective pain (*ρ* = 0.25, *p* = 0.023) and was borderline for sensory pain (*ρ* = 0.21, *p* = 0.060); emotional distress showed no significant associations. During the session, K10 scores decreased and RSES values increased, indicating immediate psychoemotional benefits. **Conclusions**: Pain perception during scaling is shaped primarily by periodontal status and psychological distress rather than by ultrasonic technology per se. Although the electronic module to dynamically adjust the power of the instrument technology may attenuate the affective component, standardized atraumatic techniques and routine psychosocial screening are likely to yield greater gains. The observed short-term improvements in distress and self-esteem support integrating patient-reported outcomes into individualized, patient-centered periodontal care.

## 1. Introduction

The preservation of oral health relies heavily on regular preventive dental examinations, which are fundamental for the early detection and prevention of periodontal disease and its associated complications. Periodontal disease represents one of the most prevalent chronic inflammatory conditions worldwide, affecting a substantial proportion of the adult population. Epidemiological data indicate that approximately 11% of adults suffer from severe periodontitis, while nearly half exhibit some degree of gingival inflammation, underscoring the urgent need for timely preventive interventions [1,2].

Scaling, a fundamental component of non-surgical periodontal therapy (NSPT), remains a cornerstone prophylactic intervention for the control of biofilms and calculus deposits. Despite its clinical benefits, scaling is frequently perceived as unpleasant or painful, an experience that may elicit dental anxiety and ultimately reduce patient compliance with preventive and therapeutic regimens [3,4].

Among patients with periodontitis, gingival inflammation and dentin hypersensitivity are frequently accompanied by heightened levels of dental anxiety and fear [5], while disease severity has been shown to correlate positively with a depressive and anxiety-related symptomatology [6,7,8]. Beyond its biological consequences, periodontitis exerts a detrimental effect on self-esteem and oral health-related quality of life [9], particularly through clinical attachment loss, tooth loss, gingival recession, esthetic impairment, and halitosis—factors that contribute to embarrassment and diminished self-confidence [6]. Accordingly, the assessment of psychological parameters such as distress and self-esteem in relation to periodontal therapy is critical for a comprehensive understanding of patients’ psychosocial recovery following treatment.

Over the past decades, significant technological advances have sought to improve both the efficacy and tolerability of scaling procedures. While manual instrumentation is often regarded as laborious and uncomfortable, ultrasonic instrumentation—based on high-frequency vibrations combined with continuous irrigation—has emerged as the current standard of care in periodontal debridement [10]. Nevertheless, conventional ultrasonic systems may still generate discomfort due to vibratory sensations and the cooling aerosol jet, particularly in patients with exposed dentin or gingival inflammation [11].

To optimize patient comfort, modern technologies have been developed, such as Device nr.1, integrated within the Guided Biofilm Therapy (GBT) concept. This system combines ultrasonic instrumentation with air-polishing and is promoted as being more tolerable for patients [12]. In contrast, Device nr.2 represents a conventional ultrasonic system. Comparative evidence regarding pain perception remains limited: some clinical studies have reported no significant differences between technologies, with both being effective and generally well accepted by patients [13]; however, contemporary air-polishing methods using glycine or erythritol powders are consistently perceived as more comfortable than conventional techniques [14].

Beyond somatic parameters, the success of periodontal therapy results in substantial psychosocial benefits by improving smile esthetics, functional comfort, and patients’ perceived control over their oral health [15,16,17]. Conversely, periodontitis constitutes a source of psychological distress—including embarrassment, fear of tooth loss, and guilt—that negatively affects self-esteem [9]. Levels of anxiety and emotional distress, measured with validated instruments such as the Rosenberg Self-Esteem Scale (RSES) and the Kessler Psychological Distress Scale (K10), influence the subjective perception of pain [18,19,20]. Evidence indicates that dental anxiety exacerbates intraoperative pain perception [21,22], even though the mean pain intensity reported during non-anesthetized scaling is relatively low [3]. More recent studies have further demonstrated a significant association between dental anxiety and pain intensity, particularly among female patients [21,23].

The primary objective of this investigation was to perform a comparative assessment, in a rigorously controlled clinical environment, of the influence exerted by the scaling technology on pain intensity, with a consideration of periodontal status and patient-specific psychological factors.

The following hypotheses were advanced:

(i) Scaling performed using an ultrasonic device featuring an electronic module to dynamically adjust the power of the instrument is associated with a lower self-reported pain intensity compared to the conventional ultrasonic device;
(ii) The periodontal status exerts a stronger influence on pain perception than the type of ultrasonic device employed;
(iii) Demographic characteristics (sex, age) exert only a minor effect on pain perception when contrasted with pathological or psychological determinants;
(iv) Baseline psychological parameters (anxiety, distress) are positively correlated with pain intensity and may exert a greater influence than the type of ultrasonic device.

By integrating both somatic and psychological dimensions, the present study seeks to inform the optimization of clinical protocols, thereby supporting the development of dental and periodontal interventions that are more effective, less traumatic, and better customized to patient-specific needs.

## 2. Materials and Methods

### 2.1. Study Design and Ethical Approval

A monocentric, split-mouth comparative clinical study was conducted and approved by the Ethics Committee of the “Iuliu Hațieganu” University of Medicine and Pharmacy, Cluj-Napoca, Romania (approval no. AVZ97/20.06.2023). The study period was June 2023–June 2025. All procedures were performed in accordance with the ethical principles of the Declaration of Helsinki (2013 revision). All patients received treatment according to their individual clinical needs, based on diagnosis, independently of their participation in this study.

The present study was a prospective, non-randomized, non-pharmacological interventional pilot clinical study carried out in a dental setting. As the interventions consisted solely of routine scaling procedures within standard clinical care, with minimal additional burden for participants (questionnaire completion), and given the minimal-risk nature of the intervention and the absence of experimental drugs or devices, formal clinical trial registration was not required according to the institutional guidelines for non-pharmacological pilot studies.

This manuscript has been prepared and reported in accordance with the TREND (Transparent Reporting of Evaluations with Nonrandomized Designs) statement, to ensure comprehensive and transparent reporting of the study design, conduct, analysis, and interpretation

### 2.2. Study Setting, Operator, and Standardization

Participants were recruited from the patient flow of the Department of Preventive Dentistry, where they attended periodic prophylaxis visits and/or non-surgical periodontal therapy. All interventions were performed by the same operator (N.C.T) to minimize inter-operator variability. Instrumental settings, clinical sequence, and materials were standardized across all subjects.

### 2.3. Participants: Eligibility and Sample

Inclusion criteria (Table 1): Individuals aged ≥18 years presenting with a permanent dentition comprising at least 20 teeth, an established indication for scaling, and the provision of signed informed consent were eligible for enrollment. Two cohorts of participants were recruited: (i) periodontally healthy subjects exhibiting gingivitis and/or supragingival calculus and (ii) patients diagnosed with stage I–III, grade B periodontitis, established through comprehensive clinical and radiographic examination in accordance with the 2017 World Workshop classification system [24].

Exclusion criteria: Patients presenting with decompensated systemic conditions (e.g., uncontrolled diabetes mellitus, severe psychiatric disorders), pregnancy, or receipt of periodontal therapy within the preceding six months were excluded.

Final sample: The study population consisted of 42 patients, of whom 21 were diagnosed with periodontitis and 21 were periodontally healthy. The cohort included 18 females and 24 males, with a mean age of 37.9 years (range: 19–65 years).

### 2.4. Devices and Operating Parameters

Two ultrasonic scaling systems were comparatively assessed:

- Airflow Prophylaxis Master (EMS, Nyon, Switzerland) (Device nr.1), employed exclusively in the piezon ultrasonic module (No Pain technology) with the air-polishing function deactivated. The EMS Piezon^®^, (no pain electronic module) incorporates a high-frequency feedback mechanism that continuously monitors changes in mechanical resistance at the instrument tip. When increased resistance indicative of calculus is detected, the device automatically adjusts and transiently increases power output to facilitate efficient deposit removal while maintaining operational stability. By contrast, standard ultrasonic scalers operate at a constant predetermined power level, without adaptive modulation in response to real-time variations in substrate resistance [12].
- EMS Piezon 250 (P250; EMS, Switzerland) (Device nr.2), a conventional piezoelectric ultrasonic device from a previous generation, not equipped with an automatic feedback system [11].

Both instruments were operated at an identical power setting of 5/10, corresponding to a medium-to-high intensity. To ensure the standardization of active instrumentation, only the standard type A tip was utilized across both devices. Continuous and abundant water irrigation was applied in conjunction with minimal lateral pressure on the tip, in strict accordance with the principles of ultrasonic instrumentation. The approximate duration of the procedure was 15–20 min per dental arch.

Chronology of the intervention and assessments (per session)

1. Patient admission and informed consent: Upon arrival at the dental clinic, patients were provided with a detailed explanation of the planned procedures, including potential risks and benefits, and subsequently signed the informed consent form.
2. Baseline psychological assessment: Prior to the intervention, psychological status was evaluated using two instruments:
  – Kessler Psychological Distress Scale (K10) [25];
  – Rosenberg Self-Esteem Scale (RSES) [26].

In the present study, we used Romanian-language versions of the Kessler Psychological Distress Scale (K10) and the Rosenberg Self-Esteem Scale (RSES). The RSES has been adapted and psychometrically evaluated in Romanian adolescent samples, showing adequate internal consistency and a coherent factorial structure [27] and it has been used as an external construct in oral-health research conducted at our institution on university students from Cluj-Napoca, where self-esteem was examined in relation to oral health-related quality of life [28]. The K10 has been incorporated as an Axis II screening tool in a doctoral study on temporomandibular disorders in a Romanian-speaking dental population, where it demonstrated adequate diagnostic performance as part of an integrated clinical protocol [29], in line with international evidence supporting the reliability and validity of this instrument across diverse clinical and community settings.

In this context, the K10 and RSES were used primarily as transdiagnostic psychoemotional indicators rather than dental-specific instruments, focusing on global psychological distress and self-esteem as key factors that may modulate patients’ pain perception and emotional responses during scaling procedures. Their internal consistency and construct validity within the present sample were further examined by exploratory factor analyses, which supported coherent underlying factor structures for both scales.

All questionnaires were self-administered under conditions that ensured privacy and confidentiality.

3. Biofilm and calculus detection: Oral biofilm was disclosed using standardized plaque disclosing agents, followed by the identification of supragingival calculus deposits through visual inspection of dried tooth surfaces and tactile examination with a dental explorer probe.
4. Maxillary scaling: Performed with Device nr.1.
  4^′^. Periodontal therapy provision: In addition to scaling, patients received periodontal therapy appropriate to their diagnosis and treatment needs, which included scaling and, when indicated, comprehensive non-surgical periodontal treatment. Pain perception questionnaires were completed immediately following the scaling procedure, after which the remaining periodontal therapy was continued. For the purpose of this study, only pain perception associated with the scaling phase was analyzed.
5. Pain assessment—maxilla: Conducted immediately after the procedure using the Short-Form McGill Pain Questionnaire (SF-MPQ).
6. Mandibular scaling: Performed with Device nr.2.
  6^′^. Periodontal therapy provision: As with the maxillary arch, patients subsequently received the periodontal therapy indicated by their diagnosis and clinical needs. Questionnaires assessing pain perception were administered immediately following scaling, and treatment was then continued. Only the perception of pain during scaling was considered in the analysis.
7. Pain assessment—mandible: Conducted immediately post-procedure using the SF-MPQ.
8. Esthetic and functional self-assessment: Following completion of the scaling procedures, patients were invited to examine their dentition in a mirror and provide a self-evaluation of esthetics and oral comfort post-treatment. Comprehensive periodontal therapy was subsequently resumed in line with the previously established treatment plan.
9. Post-intervention psychological assessment: At the conclusion of the clinical session, psychological status was re-assessed using the K10 and RSES instruments to evaluate post-treatment outcomes.

### 2.5. Sequence of Procedures

The procedural sequence was standardized and identical across all participants: maxillary scaling with Device nr.1 → pain assessment using the SF-MPQ → mandibular scaling with Device nr.2 → pain assessment using the SF-MPQ. No local or topical anesthesia was employed in order to preserve the validity and authenticity of self-reported pain measures.

### 2.6. Instruments and Scoring

- Pain assessment (SF-MPQ): Only the sensory dimension (sum of 11 descriptors; range 0–33) and the affective dimension (sum of 4 descriptors; range 0–12) were analyzed, in accordance with Melzack’s multidimensional pain model. The Visual Analog Scale (VAS, 0–100 mm) and the Present Pain Intensity (PPI, 0–5) subscales were not utilized in this investigation.
- Psychological status:
  – *RSES:* Ten items, 4-point Likert scale (total score 10–40; higher values indicate greater self-esteem).
  – *K10:* Ten items, 5-point Likert scale (total score 10–50; higher values indicate higher psychological distress).

All questionnaires were administered at baseline and immediately post-intervention within the same clinical session in a dedicated and private setting to ensure confidentiality and reduce potential bias.

### 2.7. Statistical Analysis

The present analysis was restricted exclusively to scaling (split-mouth design: Device nr.1 vs. Device nr.2), given that both periodontally healthy and periodontitis patients were included. In individuals with periodontitis, comprehensive periodontal therapy was subsequently performed in accordance with individual treatment requirements; however, these procedures were beyond the scope of the current analysis.

All data were independently double-entered into Microsoft Excel to ensure accuracy and were subsequently analyzed using SPSS Statistics, version 25.0 (IBM Corp., Armonk, NY, USA) and R, version 4.2.2 (R Foundation for Statistical Computing, Vienna, Austria). To verify construct validity and justify the derivation of composite subscale scores, exploratory factor analysis (principal axis factoring) with varimax rotation was conducted for each psychometric instrument (RSES, K10, SF-MPQ). Subscales retained included positive/negative self-esteem for RSES and sensory/affective dimensions for SF-MPQ. Factor extraction was based on the Kaiser criterion (eigenvalues > 1) and item loadings ≥ 0.40.

Descriptive statistics were computed for all variables, expressed as mean ± standard deviation (SD) or median with interquartile range (IQR), depending on distribution. Analyses were performed for the entire cohort and stratified by periodontal status (periodontitis vs. periodontally healthy). Between-group differences were evaluated using the Mann–Whitney *U* test, while paired comparisons (maxilla vs. mandible; pre- vs. post-intervention for K10 and RSES) were conducted with the Wilcoxon signed-rank test. Effect sizes were estimated using Cliff’s delta (*δ*) and interpreted according to conventional thresholds: small (~0.20), medium (~0.50), and large (~0.80).

Bivariate associations between continuous or ordinal variables were examined using Spearman’s rank correlation coefficient (*ρ*), with primary emphasis on relationships between K10 scores and SF-MPQ sensory/affective dimensions and between RSES scores and pain outcomes. Correlations between changes in psychological parameters (ΔK10 vs. ΔRSES) were also assessed. Statistical significance was defined a priori as *α* = 0.05 (two-tailed).

## 3. Results

### 3.1. Descriptive Analysis

A comprehensive descriptive analysis was undertaken for three internationally validated psychometric instruments designed to assess self-esteem, psychological distress, and pain characteristics within the study cohort (*N* = 84). The descriptive statistics reported in Table 2 summarize the central tendency, variability, and observed score distributions for each item.

On the RSES (4-point Likert, 0–3), mean item scores ranged from M = 1.74 (SD = 1.08) for the negatively valenced statement “I do not have much to be proud of” to M = 2.65 (SD = 0.70) for the positively phrased item “I feel that I have a number of good qualities.” A higher endorsement of positively worded items and a lower endorsement of negatively worded items delineated a response profile consistent with an overall favorable level of global self-esteem.

On the K10 (5-point Likert, 1–5), the pattern of responses reflected low-to-moderate psychological distress. The lowest mean score was observed for “How often did you feel worthless?” (M = 1.49; SD = 0.84), whereas a relatively higher endorsement was found for “How often did you feel nervous?” (M = 2.45; SD = 0.67). The endorsement of severe symptomatology was infrequent, exemplified by the item “So nervous that nothing could calm you down” (M = 1.53; SD = 0.57).

On the SF-MPQ (0–3 scale), the highest mean scores were recorded for the sensory descriptor “sharp/stabbing” (M = 1.20; SD = 0.98) and the affective descriptor “cruel/agonizing” (M = 0.93; SD = 1.03). The majority of other sensory and evaluative descriptors (e.g., “cramping” or “burning”) yielded mean values < 0.3, reflecting low endorsement rates.

Collectively, these findings delineate a psychometric and symptomatic profile characterized by globally favorable self-esteem, low-to-moderate psychological distress, and a generally attenuated pain intensity, with circumscribed elevations observed within the sensory dimension (stabbing/sharp pain) and affective reactivity (nervousness).

**Table 2:** Item-level descriptive statistics for the three instruments (RSES, K10, and SF-MPQ).

Item	Mean	Std. Dev.	Min.	25%	50%	75%	Max.
Rosenberg Self-Esteem Scale							
1. I feel that I am a person of worth	2.18	0.68	0	2	2	3	3
2. At times I think I am no good at all	0.65	0.69	0	0	1	1	3
3. I feel that I have a number of good qualities	2.25	0.53	0	2	2	3	3
4. I am able to do things as well as most people	2.27	0.63	0	2	2	3	3
5. I do not have much to be proud of	0.63	0.77	0	0	0	1	3
6. I feel useless at times	0.81	0.72	0	0	1	1	3
7. I feel that I’m a person of equal worth to others	2.35	0.61	0	2	2	3	3
8. I wish I could have more respect for myself	1.39	0.89	0	1	1	2	3
9. I certainly feel useless at times	0.46	0.61	0	0	0	1	2
10. I take a positive attitude toward myself	2.17	0.66	0	2	2	3	3
Kessler Psychological Distress Scale							
1. How often did you feel tired for no reason?	2.20	0.89	1	2	2	3	5
2. How often did you feel nervous?	2.45	0.67	1	2	2	3	4
3. So nervous that nothing could calm you?	1.55	0.59	1	1	1.5	2	3
4. Hopeless?	1.48	0.78	1	1	1	2	5
5. Restless or fidgety?	2.20	0.67	1	2	2	3	4
6. So restless you could not sit still?	2.11	0.74	1	2	2	2.25	5
7. Depressed?	1.58	0.70	1	1	1	2	4
8. That everything was an effort?	1.74	0.75	1	1	2	2	4
9. So depressed that nothing could cheer you up?	1.48	0.67	1	1	1	2	3
10. Worthless?	1.38	0.77	1	1	1	1.25	5
McGill Pain Questionnaire							
1. Throbbing	0.48	0.77	0	0	0	1	3
2. Excruciating (sharp stabbing)	0.93	1.03	0	0	1	2	3
3. Acute (mild stabbing)	0.76	0.91	0	0	0	1	3
4. Sharp (stinging)	1.20	0.98	0	0	1	2	3
5. Cramping	0.15	0.45	0	0	0	0	2
6. Gnawing	0.46	0.81	0	0	0	1	3
7. Burning	0.25	0.62	0	0	0	0	3
8. Persistent	0.54	0.78	0	0	0	1	3
9. Pressing	0.58	0.82	0	0	0	1	3
10. Throbbing/swollen	0.50	0.86	0	0	0	1	3
11. Cutting/tearing	0.63	0.89	0	0	0	1	3
12. Exhausting	0.31	0.68	0	0	0	0	3
13. Nauseating	0.14	0.49	0	0	0	0	3
14. Fear-inducing	0.14	0.41	0	0	0	0	2
15. Tormenting/terrible	0.27	0.61	0	0	0	0	2

**Figure 1:**
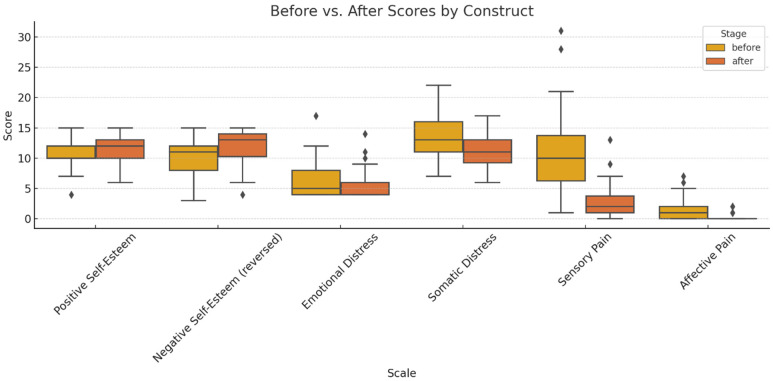
Comparative analysis of pre- and post-treatment scores across the primary psychometric dimensions (outliers marked with a rhombus).

Comparative pre–post intervention analysis (Figure 1). Items were aggregated into latent constructs: RSES (reverse-scored); K10; and SF-MPQ. This operational framework enabled an integrated and conceptually grounded interpretation of the observed pre- to post-treatment variations.

The boxplots illustrate significant improvements in self-esteem (increased scores on the positive factor and reductions in the reverse-scored negative factor) and attenuations of psychological distress (both emotional and somatic domains). The pain intensity, particularly within the sensory dimension, declined post-intervention, with parallel reductions observed in affective descriptors (exhausting, fearful, distressing). Collectively, these findings underscore a holistic multidimensional benefit—cognitive–emotional, psychological, and somatic—culminating in enhanced subjective well-being.

### 3.2. Results of Factor Analysis

**Figure 2:**
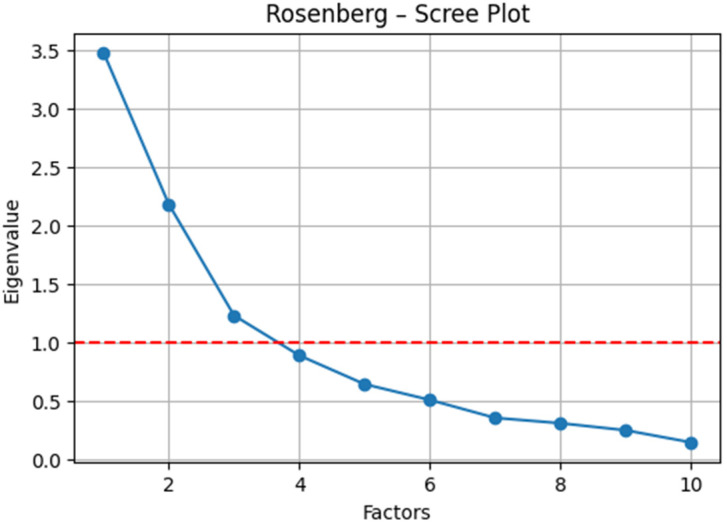
Eigenvalue scree plot for the RSES (cut-off value marked with a red line).

RSES (Figure 2): The exploratory factor analysis (varimax rotation) conducted on the pre-intervention data indicated sampling adequacy (KMO = 0.65) and supported factorability (Bartlett’s test of sphericity: *χ*^2^ (45) = 165.12, *p* < 0.001). The two-factor solution (Table 3) delineated a negative self-evaluation factor (high loadings on negatively worded items, e.g., 0.86, 0.82, and 0.68) and a positive self-esteem factor (loadings on positively worded items, e.g., 0.69, 0.60, and 0.96), thereby reproducing the canonical bifactorial structure.

**Figure 3:**
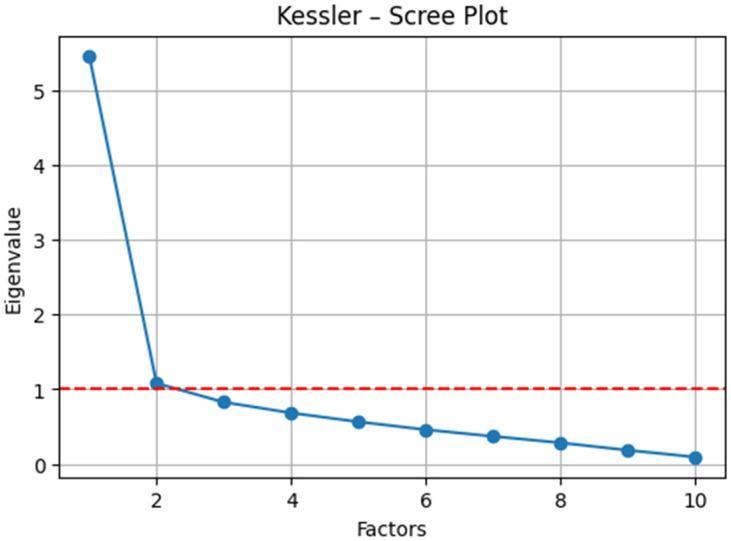
Eigenvalue scree plot illustrating the factor structure of the K10 (cut-off value marked with a red line).

K10 (Figure 3): The sampling adequacy was high (KMO = 0.79), and Bartlett’s test of sphericity confirmed the suitability of the data for factor analysis (*χ*^2^ (45) = 237.86, *p* < 0.001). The exploratory factor analysis (principal axis factoring with varimax rotation) revealed a robust two-factor solution (Table 4): (i) somatic tension/agitation (e.g., fatigue without reason, loading = 0.70; restlessness, loading = 0.74; and “everything was an effort,” loading = 0.68), and (ii) affective–cognitive distress (e.g., hopelessness, loading = 0.77; worthlessness, loading = 0.86; and severe depression, loading = 0.68). This factorial configuration substantiates the multidimensional construct of psychological distress and aligns with theoretical expectations for the K10.

**Figure 4:**
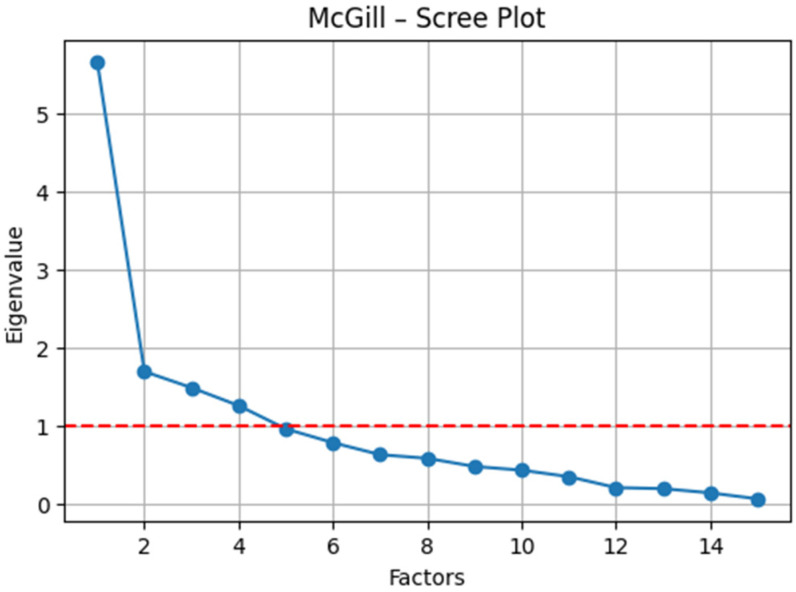
Eigenvalue scree plot illustrating the factor structure of the SF-MPQ (cut-off value marked with a red line).

McGill Pain Questionnaire (SF-MPQ) (Figure 4): The sampling adequacy was acceptable (KMO = 0.67), and Bartlett’s test of sphericity confirmed factorability (*χ*^2^ (105) = 303.20, *p* < 0.001). The exploratory factor analysis supported a bifactorial structure (Table 5): (i) sensory pain (e.g., cramping, loading = 0.69; gnawing, loading = 0.79; persistent, loading = 0.72; and pressing, loading = 0.70) and (ii) affective/evaluative pain (e.g., sharp, loading = 0.80; excruciating, loading = 0.55; and nauseating, loading = 0.41). Items with a mixed valence (e.g., “terrible,” “fear-provoking”) demonstrated moderate cross-loadings on both factors, reflecting the inherent sensory–emotional interplay of the nociceptive experience.

### 3.3. Descriptive and Graphical Analysis of Factor Scores

**Figure 5:**
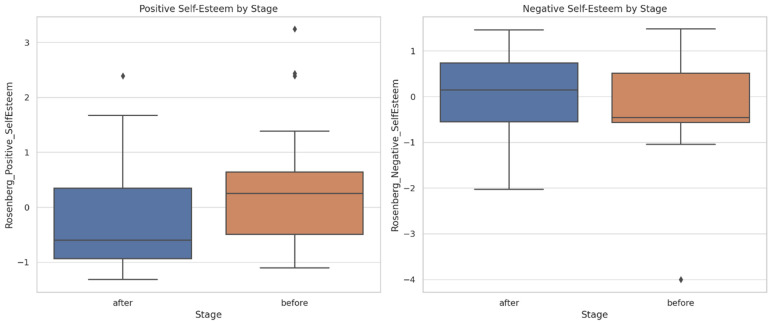
Pre- and post-treatment self-esteem scores of participants as measured by the RSES (outliers marked with a rhombus).

Rosenberg Scale (Figure 5): Positive self-esteem increased from pre- to post-intervention, while negative self-esteem (reverse-scored) decreased—indicating an overall improvement in self-perception following scaling.

**Figure 6:**
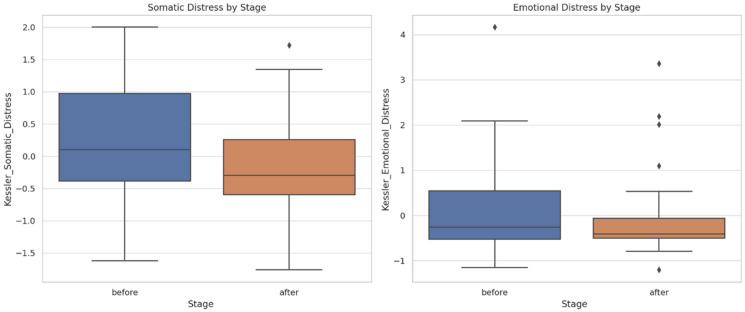
Pre- and post-treatment psychological distress scores of patients (K10); outliers marked with a rhombus.

Kessler Scale (Figure 6): Somatic and emotional distress were elevated at baseline and demonstrated a post-intervention decline, suggesting potential psychological benefits attributable to dental treatment, including reductions in discomfort, anxiety, and esthetic-related stigma.

**Figure 7:**
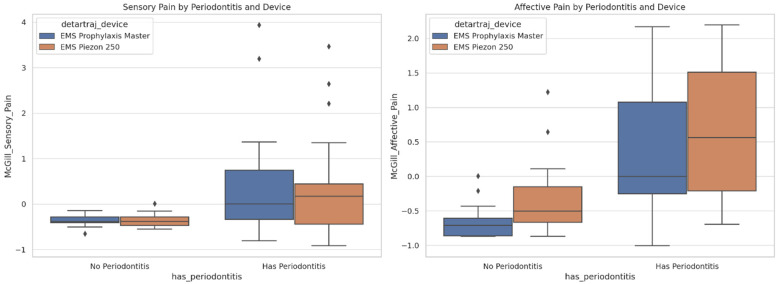
Pre- and post-treatment pain perception scores of participants (SF-MPQ); outliers marked with a rhombus.

McGill Pain Perception (Figure 7): Sensory and affective pain scores were markedly elevated in patients with periodontitis relative to periodontally healthy individuals, irrespective of the ultrasonic device utilized (device nr.2 vs. device nr.1). In non-periodontitis patients, scores clustered around zero, reflecting negligible discomfort. The inter-device variability was minimal, underscoring the periodontal status as the predominant determinant of nociceptive experiences.

The descriptive and graphical findings suggest post-treatment improvements in self-esteem and psychological well-being, while concurrently demonstrating elevated pain levels among patients with periodontitis. The statistical significance of these differences was subsequently evaluated through rigorous hypothesis-testing procedures.

### 3.4. Hypothesis Testing

1. Device nr.1 vs. device nr.2.

The Mann–Whitney U analysis revealed no significant differences in sensory pain (*U* = 909.00, *p* = 0.597), whereas affective pain was significantly lower with the Device nr.1 (*U* = 644.00, *p* = 0.017), indicating a potential affective benefit without a corresponding sensory effect.

2. Absence of differences between technologies.

The two-tailed Mann–Whitney *U* analysis confirmed non-significant differences in sensory pain (*U* = 909.00, *p* = 0.813) but identified a significant difference in affective pain (*U* = 644.00, *p* = 0.034), suggesting that the affective dimension of pain may be more sensitive to the type of ultrasonic technology employed.

3. Periodontitis vs. non-periodontitis—higher pain levels.

Mann–Whitney U tests demonstrated significantly higher scores in patients with periodontitis for both sensory (*U* = 509.00, *p* = 0.0004) and affective pain (*U* = 290.00, *p* < 0.0001), irrespective of the device employed.

4. Periodontal involvement outweighs technology in predicting pain.

The effect size analysis (Cliff’s *δ*) indicated a moderate-to-strong effect of periodontitis on sensory pain (*δ* = 0.42) and a large effect on affective pain (*δ* = 0.67). In contrast, the type of ultrasonic device exerted a negligible effect on sensory pain (*δ* = −0.03) and only a small effect on affective pain (*δ* = 0.27). The results demonstrate that periodontal status constitutes a stronger determinant of pain perception than the ultrasonic technology employed, exerting significant effects on both sensory and affective dimensions.

5. Psychological status outweighs technology in pain perception.

Spearman’s correlations indicated a significant association between somatic distress and affective pain (*ρ* = 0.25, *p* = 0.023) and a marginal association with sensory pain (*ρ* = 0.21, *p* = 0.060). Emotional distress showed no significant relationship with either pain dimension. In comparison, effect sizes for technology were negligible for sensory pain (*δ* = −0.03) and small for affective pain (*δ* = 0.27). These findings suggest that psychological distress—particularly its somatic component—more strongly modulates the nociceptive experience than the type of ultrasonic technology employed (Table 6).

## 4. Discussion

At the outset of this discussion, it should be emphasized that the null hypothesis was partially rejected. Significant differences between ultrasonic devices were identified only for the affective dimension of pain, whereas sensory pain remained comparable across technologies. The results therefore indicate that the type of ultrasonic scaler exerted a limited influence on the nociceptive experience. In contrast, periodontal status and psychological distress emerged as the primary determinants of pain perception, exerting markedly stronger effects than the technological variable.

### 4.1. Comparative Assessment of Ultrasonic Technologies

The present study comparatively evaluated patient comfort during scaling performed with two ultrasonic technologies while accounting for periodontal status. Both devices were well tolerated, with low mean pain intensity, aligning with evidence that recent advancements in prophylactic instrumentation do not fully eliminate discomfort [13,22]. Despite manufacturers’ claims regarding the superiority of the automated pressure-regulation ultrasonic system, no clinically meaningful differences were observed compared with conventional ultrasonics under standardized conditions. These findings reinforce the predominance of operator proficiency and atraumatic technique as key determinants of patient comfort [13,22].

### 4.2. Influence of Periodontal Status on Pain Perception

Patients with periodontitis reported significantly higher sensory and affective pain scores than periodontally healthy individuals, consistent with literature linking gingival inflammation, root hypersensitivity, and emotional burden to amplified nociception [22,30,31]. Clinically, these results highlight the potential need for desensitizing measures or local anesthesia in patients predicted to experience heightened sensitivity [21,32,33].

### 4.3. Psychological Determinants of Pain Experience

Emotional factors substantially influenced pain perception, confirming evidence that dental procedures associated with anticipatory anxiety intensify stress and modulate nociceptive responses [3,34,35]. Although self-esteem (RSES) did not directly correlate with pain, its inverse relationship with psychological distress (K10) suggests an indirect psychosocial pathway.

### 4.4. Multidimensional Perspective and Clinical Implications

The findings collectively indicate that periodontal status is the principal determinant of pain perception, whereas ultrasonic device type exerts a limited effect. Improvements in psychological measures (decreased K10 and increased RSES) after treatment highlight the short-term psychological benefits of scaling, suggesting that treatment outcomes should consider both clinical and psychological parameters.

### 4.5. Methodological Limitations and Future Directions

Fixed device allocation (Device nr.1 for the maxilla and Device nr.2 for the mandible) and constant treatment order may have introduced arch- or order-related bias, although the split-mouth design mitigated inter-individual variation. The lack of operator and patient blinding could have generated expectancy effects, yet no consistent directional influence was observed. The air-polishing function of device nr.1 was not used, limiting conclusions to ultrasonic scaling, despite previous reports of increased comfort with subgingival powder application [14]. Operator comfort, noted as relevant in prior research [13], was not evaluated. Future studies should examine the performance of the automated pressure-regulation ultrasonic system in periodontal pocket debridement, compare air-polishing and ultrasonic modalities within the Guided Biofilm Therapy framework, and develop predictive models integrating technological, periodontal, and psychological contributors to pain perception.

A further limitation pertains to the psychometric instruments used in this study. Although the K10 and the RSES have demonstrated acceptable properties in Romanian samples [27,28,29], these instruments were not originally designed for dentistry-specific applications and have not undergone comprehensive validation within Romanian dental populations. Consequently, the present findings should be interpreted as reflecting broader psychoemotional correlates of dental pain rather than constructs uniquely anchored in dental settings. Future investigations are encouraged to employ, adapt, or develop psychometric tools that are fully validated and tailored to dentistry-specific contexts.

## 5. Conclusions

1. Low pain intensity during scaling procedures.

Scaling performed without anesthesia was generally characterized by a low level of perceived pain, particularly among patients without an active periodontal pathology. This finding confirms that modern ultrasonic instrumentation enables the efficient removal of dental deposits with minimal discomfort—an observation of direct clinical relevance for demystifying the popular perception that scaling is inherently painful and for improving compliance with routine prophylaxis.

2. Technological equivalence under standardized conditions.

Comparisons between Device nr.1 and Device nr.2 revealed no significant differences in perceived pain levels when operative parameters were standardized. This suggests that the technological advancement of the electronic module to dynamically adjust the power of the instrument does not translate into clinically perceptible nociceptive benefits.

3. Impact of periodontal status on pain experience.

Periodontal status emerged as the principal determinant of pain perception. Patients with periodontitis reported significantly higher levels of both sensory and affective pain compared with periodontally healthy patients, irrespective of the ultrasonic device used. These results confirm that gingival inflammation and tissue alterations associated with periodontal disease are major contributors to heightened discomfort during scaling.

4. Psychological determinants of pain perception.

The findings highlighted a significant association between levels of anxiety/distress and pain intensity. Patients with anxious psychological profiles reported more intense and affectively negative experiences, corroborating the literature that documents the interdependence between dental anxiety and nociception. This underscores the importance of integrating psychological assessments into routine practice—whether through informal inquiry or validated instruments (e.g., MDAS, K10)—and of implementing supportive psychological interventions (empathetic communication, reassurance, and fostering a sense of control), which may optimize the patient experience to a degree comparable with technological innovations.

5. Immediate psychological benefit of the procedure.

A novel contribution of the present study was the identification of an immediate psychological benefit associated with scaling. The significant reduction in distress levels (K10 scores) alongside the concomitant increase in self-esteem (RSES scores) post-intervention reflect not only the attenuation of anticipatory anxiety but also a genuine gain in psychological well-being and self-perception. These findings support the integration of scaling into an expanded paradigm in which therapeutic success is defined not solely by objective clinical parameters but also by its impact on patients’ quality of life and emotional balance.

6. Clinical implications and practical recommendations.

The results indicate that pain perception during scaling is predominantly influenced by the periodontal status and patients’ anxiety levels rather than by the type of ultrasonic technology employed. Consequently, preventive and therapeutic strategies should be individualized by identifying patients at higher risk of discomfort (particularly those with periodontitis and/or elevated anxiety) and tailoring treatment protocols accordingly. With regard to equipment selection, the data suggest that procedural tolerability remains comparable when operator parameters are standardized. Therefore, the choice of ultrasonic device may be based on pragmatic considerations such as availability, ergonomics, or cost, without significantly affecting perceived patient comfort.

### General Conclusions

The findings of this study demonstrate that nociceptive perception associated with scaling is primarily determined by periodontal status and the patient’s psychological profile, surpassing the relevance of the technological determinant represented by the type of ultrasonic device employed. Furthermore, this investigation is the first to document an immediate psychological benefit of this prophylactic procedure, evidenced by a reduction in distress levels and an improvement in self-esteem, thereby conferring a multidimensional impact that transcends the strictly somatic domain. By simultaneously integrating objective clinical parameters and subjective psychological variables within the framework of a routine therapeutic intervention, this study distinguishes itself through an undeniable originality and innovative contribution to the scientific literature. These findings support a reconceptualization of dental prophylaxis, wherein the success of the treatment is defined not solely by the efficiency of the calculus removal but also by its contribution to psychological well-being and the enhancement of patients’ quality of life, thereby reinforcing the foundation of a holistic and personalized paradigm in contemporary dental medicine.

## Figures and Tables

**Table 1:** Inclusion and exclusion criteria.

Inclusion Criteria	Exclusion Criteria
Age ≥ 18 years	Decompensated systemic conditions (e.g., uncontrolled diabetes mellitus, cardiovascular disease)
Permanent dentition with ≥20 teeth	Pregnancy or lactation
Indication for scaling	Severe psychiatric disorders or inability to complete questionnaires
Provision of written informed consent	Periodontal therapy received within the previous 6 months
Ability to attend both study sessions	Current use of analgesic, anti-inflammatory, or anxiolytic medication

**Table 3:** Factor loading matrix of the RSES items, evidencing the bifactorial construct of positive and negative self-esteem.

Item	Factor 1	Factor 2
I am generally satisfied with myself	−0.58	−0.03
At times I think I am no good at all	0.86	0.04
I feel that I have a number of good qualities	−0.21	0.69
I am able to do things as well as most people	−0.08	0.6
I feel I do not have much to be proud of	0.82	−0.21
I feel useless at times	0.68	−0.27
I feel that I’m a person of equal worth to others	0.08	0.96
I wish I had more respect for myself	0.43	0.25
I tend to think I’m a failure	0.38	−0.07
I have a positive attitude toward myself	−0.47	0.25

**Table 4:** Factor loading matrix of the K10 items, delineating the two-factor structure of somatic tension/agitation and affective–cognitive distress.

Item	Factor 1	Factor 2
How often did you feel tired for no reason?	0.7	0.2
How often did you feel nervous?	0.46	0.32
How often did you feel so nervous that nothing could calm you down?	0.54	0.2
How often did you feel hopeless?	0.43	0.77
How often did you feel restless or fidgety?	0.63	0.3
How often did you feel so restless you could not sit still?	0.74	0.38
How often did you feel depressed?	0.46	0.67
How often did you feel that everything was an effort?	0.68	0.36
How often did you feel so depressed that nothing could cheer you up?	0.22	0.68
How often did you feel worthless?	0.33	0.86

**Table 5:** Factor loading matrix of the SF-MPQ items, evidencing the bifactorial structure of sensory and affective/evaluative pain.

Item	Factor 1	Factor 2
throbbing/pulsing	0.52	0.36
excruciating (severe stabbing)	0.42	0.55
acute (milder stabbing)	0.27	0.8
sharp (pricking)	0.34	0.32
cramping	0.69	0.08
gnawing	0.79	0.07
burning (searing)	0.61	0.15
persistent/annoying	0.72	0.32
pressing	0.7	0.15
swelling (painful)	0.37	0.55
cutting (tearing)	0.49	0.25
exhausting	0.4	0.48
nauseating	−0.24	0.41
fear-inducing	0.05	0.34
tormenting/terrible	0.38	0.41

**Table 6:** Results of correlation analysis and effect size estimation.

Predictor Variable	Outcome Variable	Effect Size Type	Value	*p*-Value
Somatic Distress	Sensory Pain	Spearman *ρ*	0.206	0.06
Somatic Distress	Affective Pain	Spearman *ρ*	0.247	0.023 *
Emotional Distress	Sensory Pain	Spearman *ρ*	0.121	0.272
Emotional Distress	Affective Pain	Spearman *ρ*	0.142	0.196
Technology Used	Sensory Pain	Cliff’s *δ*	−0.03	—
Technology Used	Affective Pain	Cliff’s *δ*	0.27	—

* *p* < 0.05.

## Data Availability

The datasets generated and analyzed during the current study are not publicly available due to ethical and privacy restrictions.

## Abbreviations

The following abbreviations are used in this manuscript:

NSPT	Non-Surgical Periodontal Therapy
GBT	Guided Biofilm Therapy
RSES	Rosenberg Self-Esteem Scale
K10	Kessler Psychological Distress Scale
SF-MPQ	Short-Form McGill Pain Questionnaire
VAS	Visual Analog Scale
PPI	Present Pain Intensity
SD	Standard Deviation
IQR	Interquartile Range
KMO	Kaiser–Meyer–Olkin Measure of Sampling Adequacy
χ^2^	Chi-Square Test
ρ (rho)	Spearman’s Rank Correlation Coefficient
δ (delta)	Cliff’s Delta (Effect Size)
